# AGE-RELATED DIFFERENCES IN KETONE METABOLISM: A STUDY OF KETONE ESTER RESPONSES ACROSS LIFESPAN

**DOI:** 10.1093/geroni/igae098.3634

**Published:** 2024-12-31

**Authors:** Ester Hernandez, Aydan Jordan, Chatura Senadheera, Elizabeth Stephens, John Newman, Madison Kackley, Jeff Volek, Brianna Stubbs

**Affiliations:** Buck Institute for research on aging, Cotati, California, United States; Ohio State University, Columbus, Ohio, United States; Buck Institute, Novato, California, United States; Buck Institute for Research on Aging, Novato, California, United States; Buck Institute for Research on Aging, Novato, California, United States; Ohio State University, Columbus, Ohio, United States; Ohio State University, Columbus, Ohio, United States; Buck Institute, Novato, California, United States

## Abstract

Ketone supplements, such as ketone esters, induce ketosis without other dietary changes and have demonstrated many potential applications in younger adults. However, metabolism by older adults is unknown. This study investigated the metabolic responses to ketone ester consumption across the lifespan, to understand how aging influences blood ketone responses. This study was an open-label, single-arm, observational study of n=82 adults (37=Male, age=20-70y). After an overnight fast, subjects consumed 360 mg/kg/lean body mass of ketone ester (bis-octanoyl-(R)-1,3-butanediol) in a beverage. Then, capillary blood samples were collected at regular intervals for 4h and beta-hydroxybutyrate (BHB) was assayed using a handheld device. Endpoints were peak BHB concentration (Cmax), area under the curve (AUC). The analysis of BHB Cmax across five decades showed no overall effect (p=0.5), with the highest Cmax in the 41-50y group (1.68 mM) and the lowest in the 51-60y group (1.32 mM). In contrast, although there was no overall difference in BHB AUC between age groups (p=0.24, mean mm.min-1, age 20-30y =184, 31-40y =194, 41-50y =221, 51-60y =203, 61-70y =223) there was a notable difference between the 21-30y and 61-70y groups (p=0.03). Linear regression analysis revealed a positive relationship between BHB AUC and age (R²=0.08, p=0.01), but not between BHB Cmax and age (R²=0.01, p=0.37). We cautiously interpret our data to suggest a relationship between increasing age and changes to ketone metabolism that increases AUC but not Cmax. We plan to expand the sample size to 400 subjects to improve our ability to detect-age related differences in exogenous ketone metabolism.

